# Effects of New Bayesian Penalized Likelihood Reconstruction Algorithm on Visualization and Quantification of Upper Abdominal Malignant Tumors in Clinical FDG PET/CT Examinations

**DOI:** 10.3389/fonc.2021.707023

**Published:** 2021-08-16

**Authors:** Mitsuaki Tatsumi, Fumihiko Soeda, Takashi Kamiya, Junpei Ueda, Daisuke Katayama, Keiko Matsunaga, Tadashi Watabe, Hiroki Kato, Noriyuki Tomiyama

**Affiliations:** ^1^Department of Radiology, Osaka University Hospital, Suita, Japan; ^2^Department of Nuclear Medicine and Tracer Kinetics, Osaka University Graduate School of Medicine, Suita, Japan; ^3^Department of Medical Technology, Osaka University Hospital, Suita, Japan

**Keywords:** Bayesian penalized likelihood reconstruction, PET, quantitative imaging, abdomen, malignant tumor

## Abstract

**Purpose:**

This study evaluated the effects of new Bayesian penalized likelihood (BPL) reconstruction algorithm on visualization and quantification of upper abdominal malignant tumors in clinical FDG PET/CT examinations, comparing the results to those obtained by an ordered subset expectation maximization (OSEM) reconstruction algorithm. Metabolic tumor volume (MTV) and texture features (TFs), as well as SUV-related metrics, were evaluated to clarify the BPL effects on quantification.

**Materials and Methods:**

A total of 153 upper abdominal lesions (82 liver metastatic and 71 pancreatic cancers) were included in this study. FDG PET/CT images were acquired with a GE Discovery 710 scanner equipped with a time-of-flight system. Images were reconstructed using OSEM and BPL (beta 700) algorithms. In 58 lesions <1.5 cm in greatest diameter (small-lesion group), visual image quality of each lesion was evaluated using a four-point scale. SUVmax was obtained for quantitative metrics. Visual scores and SUVmax were compared between OSEM and BPL images. In 95 lesions >2.0 cm in greatest diameter (larger-lesion group), SUVmax, SUVpeak, MTV, and six TFs were compared between OSEM and BPL images. In addition to the size-based analyses, an increase of SUVmax with BPL was evaluated according to the original SUVmax in OSEM images.

**Results:**

In the small-lesion group, both visual score and SUVmax were significantly higher in the BPL than OSEM images. The increase in visual score was observed in 20 (34%) of all 58 lesions. In the larger-lesion group, no statistical difference was observed in SUVmax, SUVpeak, or MTV between OSEM and BPL images. BPL increased high gray-level zone emphasis and decreased low gray-level zone emphasis among six TFs compared to OSEM with statistical significance. No statistical differences were observed in other TFs. SUVmax-based analysis demonstrated that BPL increased and decreased SUVmax in lesions with low (<5) and high (>10) SUVmax in original OSEM images, respectively.

**Conclusion:**

This study demonstrated that BPL improved conspicuity of small or low-count upper abdominal malignant lesions in clinical FDG PET/CT examinations. Only two TFs represented significant differences between OSEM and BPL images of all quantitative metrics in larger lesions.

## Introduction

Ordered subset expectation maximization (OSEM) iterative algorithm has been used for image reconstruction in positron emission tomography (PET) imaging. However, OSEM is known to have a limitation in quantification as it stopped before reaching full convergence due to image noise increased with each iteration. This compromise result in providing insufficient quantitative values.

Bayesian penalized likelihood (BPL) reconstruction algorithm, or so-called “Q. Clear”, was recently supplied by GE Healthcare to improve both the image quality and quantification in PET examinations (1). Although BPL theoretically affects visual images and quantitative values, little is known about their changes in real clinical situations. There have been several reports regarding the effects of BPL in pulmonary lesions (2–6). Only limited reports have been available so far as to the effects of BPL focusing on abdominal lesions in 2-deoxy-2-[F-18]fluoro-D-glucose (FDG) PET imaging (7). Clear visualization of upper abdominal lesions is often challenging as they move according to patients’ respiratory motion. The visualization of liver lesions is also hindered by background FDG activity in normal liver, which represents diffuse mild to moderate uptake. As for pancreatic cancer lesions, mild FDG uptake is often observed due to abundant fibrous components with scattered cancer cells (8, 9), although the cancer is one of the major malignancies examined with FDG PET/computed tomography (CT) examinations in the abdominal region. BPL is expected to improve the visualization and detection of these abdominal lesions as well as pulmonary lesions.

In the present study, we evaluated the effects of this new algorithm on visualization and quantification of upper abdominal malignant tumors in FDG PET/CT examinations, comparing the results to those obtained by an OSEM reconstruction algorithm. Metabolic tumor volume (MTV) and texture features (TFs), as well as SUV-related metrics, were evaluated to clarify the BPL effects on quantification.

## Materials and Methods

A total of 153 upper abdominal malignant lesions evaluated with FDG PET/CT was included in this study. The abdominal malignant lesions, proven by biopsy or imaging follow-up, consisted of 82 lesions of metastatic liver cancer and 71 lesions of pancreatic cancer. There were 58 and 95 abdominal malignant lesions less than 1.5 cm (34 liver and 24 pancreatic lesions) and more than 2.0 cm (48 liver and 47 pancreatic lesions) in greatest diameter, respectively, which were designated as small and larger lesions. No lesions between 1.5 and 2.0 cm in the greatest diameter were included in this study.

FDG PET/CT imaging was performed with a GE Discovery 710 scanner equipped with a time-of-flight system. PET images were acquired for 2 min per one bed position 60 min after intravenous injection of FDG at a dose of 0.10 mCi/kg body weight. The PET field of view was 70 cm. They were reconstructed using OSEM (subset 8, iteration 3, and Gaussian filter 4 mm; regular setting in our hospital) and BPL with beta 700 algorithms, as well as time-of-flight and point spread function. All PET images were reconstructed with 192 × 192 matrix.

The beta value 700 was determined according to our previous study of PET image quality with BPL reconstruction algorithm using several beta values (10). PET images reconstructed using OSEM and BPL were compared in the phantom and clinical studies, and BPL images with beta 700 exhibited better recovery coefficient and signal-to-noise ratio values than OSEM images regularly acquired in our hospital.

### The Effect of BPL in Small Lesions

This group included 58 upper abdominal malignant (34 liver metastatic and 24 pancreatic cancer) lesions less than 1.5 cm in greatest diameter.

PET images reconstructed using OSEM and BPL (hereinafter, OSEM and BPL images, respectively) were interpreted visually by two board-certificated nuclear medicine physicians/radiologists (both having double board certifications) separately, and lesion conspicuity was evaluated with a four-point scale (1: poor, 2: fair, 3: good, and 4: excellent). Scores were recorded, and discrepancies, if any, were resolved by consensus between the two observers.

The maximum value of standardized uptake value (SUVmax) was obtained for quantitative metrics. SUVmax was calculated from a single voxel exhibiting the maximum SUV in each lesion. Other quantitative results were not obtained as the results were unreliable due to their small lesion sizes.

### The Effect of BPL on Quantification in Larger Lesions

This group included 95 lesions (48 liver and 47 pancreatic lesions) more than 2 cm in greatest diameter.

SUVmax, SUVpeak, and MTV were compared between OSEM and BPL images in each lesion. SUVpeak was mean SUV of a 1 cm^3^ three-dimensional region-of-interest covering the area with most intense FDG uptake in the same lesion where SUVmax was calculated (11). MTV was defined as the volume within a tumor margin, which was delineated with 40% of SUVmax. These quantitative metrics were obtained with commercially available software (PETSTAT: AdIn Research, Tokyo, Japan).

Texture features, quantitative indicators of intratumoral heterogeneity, were also compared in this group. Texture features evaluated in this study were selected according to the report by Orlhac et al. (12). Homogeneity and entropy were calculated from the co-occurrence matrix, short-run emphasis (SRE) and long-run emphasis (LRE) from the gray-level run length matrix, and low gray-level zone emphasis (LGZE) and high gray-level zone emphasis (HGZE) from the gray-level zone length matrix. These six texture features were reported to be the most robust with respect to tumor region delineation and relatively independent one from another and were obtained with the same software as described above (PETSTAT). Homogeneous lesions are known to have higher values of homogeneity and LGZE and lower values of entropy and HGZE than visually heterogeneous lesions (12).

### SUV-Based Analysis

In addition to analyses in the subgroups based on lesion sizes, the difference of increase in SUVmax with BPL was analyzed according to the original SUVmax in OSEM. This analysis was conducted in all small and larger lesions.

Low, intermediate, and high SUVmax were defined as SUVmax less than 5, 5 to 10, and more than 10 in OSEM images, respectively. The increase in SUVmax with BPL was evaluated in lesions with low, intermediate, and high SUVmax in OSEM images.

### Statistical Analysis

Visual scores and quantitative metrics were compared between OSEM and BPL with a Wilcoxon signed-rank test. Correlations were analyzed with a Spearman’s rank method. A P value less than 0.05 was considered statistically significant. Data were expressed as mean ± standard deviation.

## Results

### The Effect of BPL in Small Lesions

Visual analysis demonstrated that the score was 2.8 ± 0.97 in OSEM and 3.2 ± 0.82 in BPL images. The score in BPL was significantly higher than that in OSEM images (p < 0.0001). The visual score was increased with BPL in 20 (33%) of all 58 lesions (3 lesions: 1 in OSEM to 2 in BPL, 11 lesions: 2 in OSEM to 3 in BPL, 1 lesion: 2 in OSEM to 4 in BPL, and 5 lesions: 3 in OSEM to 4 in BPL) (**Table 1**).

**Table 1:** Number of lesions according to score in PET images with OSEM and BPL reconstruction.

Score in OSEM	Score in BPL					
	1	2	3	4		
1	1	3	0	0	(4)	
2	0	10	11	1	(22)	
3	0	0	10	5	(15)	
4	0	0	0	17	(17)	
	(1)	(13)	(21)	(23)		

Parentheses indicate total number in each row or column.

OSEM, ordered subset expectation maximization; BPL, Bayesian penalized likelihood.

**Figure 1:**
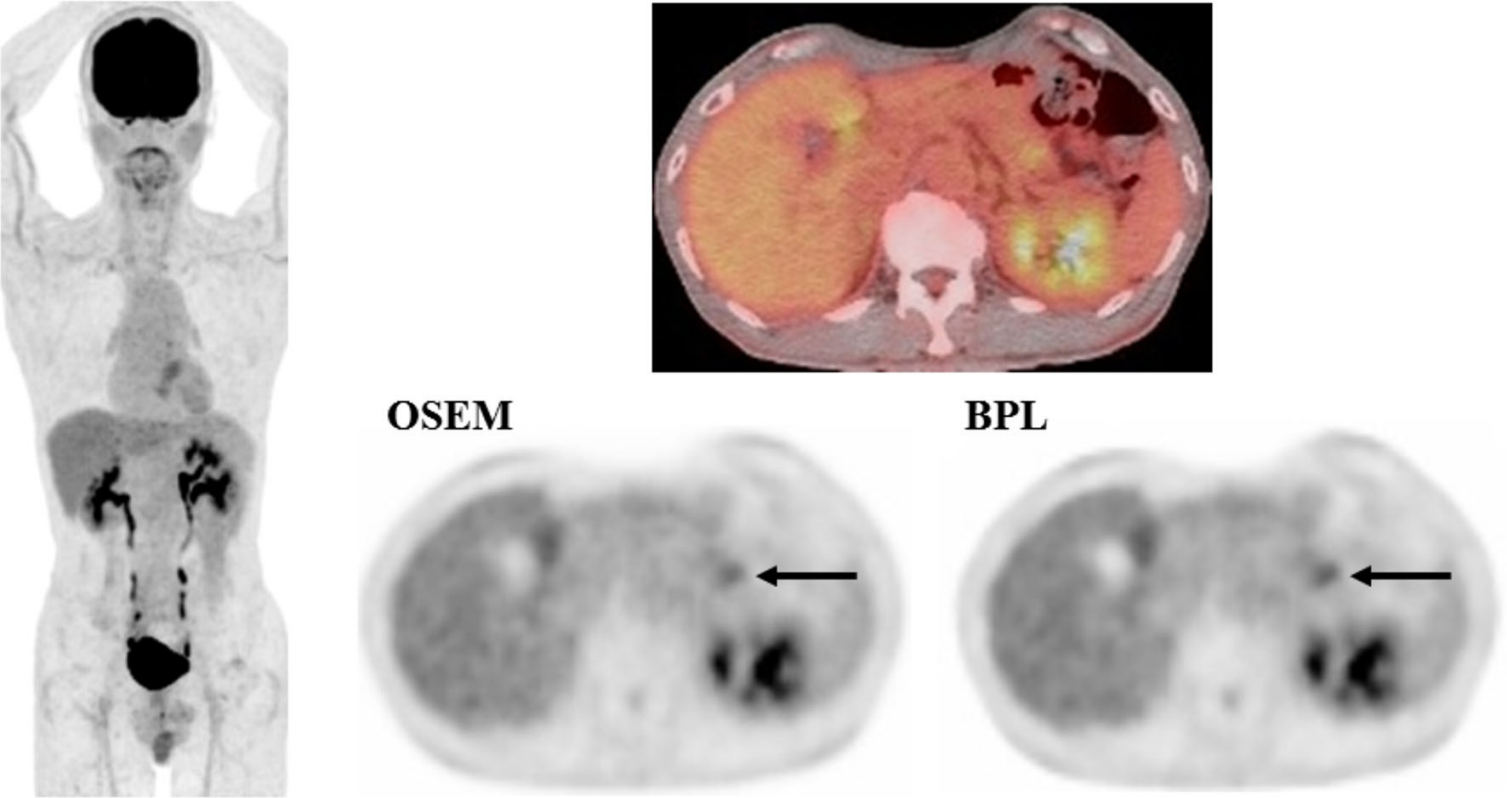
A case with pancreatic cancer (small lesion). FDG uptake corresponding to the pancreatic cancer lesion (arrow) was observed more clearly in BPL than OSEM image (right, lower). SUVmax was higher in BPL (2.9) than in OSEM image (2.7). (Left: maximum intensity projection image of FDG PET; right upper: fused FDG PET/CT image; right lower: FDG PET images reconstructed with OSEM and BPL algorithms.).

SUVmax represented the same trend as the visual score. SUVmax was 4.45 ± 2.4 in OSEM and 4.63 ± 2.4 in BPL images. SUVmax in BPL was significantly higher than that in OSEM images (p<0.05) (5.9% increase with BPL on average). A case with small pancreatic cancer is presented in **Figure 1**, which demonstrated that BPL improved lesion conspicuity compared with OSEM images.

The increase in visual score with BPL showed a negative correlation with the score in OSEM or SUVmax in OSEM images (|Rho| = 0.51–0.55, p<0.0001), and positive correction with %increase of SUVmax with BPL (Rho = 0.52, p<0.0001).

### The Effect of BPL in Larger Lesions

In this group of 95 lesions, tumor size ranged from 20 to 98 mm (36.9 ± 16.8 mm). SUVmax, SUVpeak, and MTV were 10.1 ± 4.0, 8.1 ± 3.2, and 32.8 ± 64.3 ml in OSEM images, and 9.9 ± 3.9, 8.2 ± 3.2, and 37.8 ± 84.7 ml in BPL images, respectively.

BPL increased SUVpeak and MTV by 1.4 and 7.0%, respectively, and decreased SUVmax by 1.0% compared to OSEM as on average. However, no statistical difference was observed in SUVmax, SUVpeak, or MTV between OSEM and BPL images (**Table 2**).

**Table 2:** SUV, MTV, and texture features in PET images with OSEM and BPL reconstruction.

	OSEM	BPL	%increase
SUVmax	10.1 ± 4.0	9.9 ± 3.9	−1.0 ± 3.0
	(4.6–17.3)	(4.6–16.6)	(-8.5–5.8)
SUVpeak	8.1 ± 3.2	8.2 ± 3.2	1.4 ± 2.7
	(4.2–14.4)	(4.2–14.3)	(−4.0–7.8)
MTV	32.8 ± 64.3	37.8 ± 84.7	7.0 ± 15.0
	(0.9–308)	(1.1–418)	(−30.5–39.5)
Homogeneity	0.16 ± 0.05	0.16 ± 0.06	1.1 ± 6.4
	(0.09–0.38)	(0.11–0.39)	(−12.9–14.6)
Entropy	5.0 ± 1.2	5.0 ± 1.3	1.1 ± 5.8
	(1.9–7.0)	(2.3–6.9)	(−17.4–16.6)
Short-run emphasis	0.98 ± 0.01	0.98 ± 0.02	−0.02 ± 0.96
	(0.93–1.0)	(0.91–1.0)	(−2.3–2.7)
Long-run emphasis	1.1 ± 0.08	1.1 ± 0.09	0.05 ± 3.2
	(1.0–1.4)	(1.0–1.5)	(−9.5–6.3)
Low gray-level zone emphasis	0.07 ± 0.03	0.05 ± 0.02^*^	−13.3 ± 18.6
	(0.02–0.16)	(0.02–0.11)	(−70.9–22.6)
High gray-level zone emphasis	565 ± 162	644 ± 201^*^	13.9 ± 17.0
	(72–885)	(72–1091)	(−2.3–67)

SUV, standardized uptake value; SUVmax, maximum value of SUV; SUVpeak, peak value of SUV; MTV, metabolic tumor volume; OSEM, ordered subset expectation maximization; BPL, Bayesian penalized likelihood.

Parentheses indicate range.

*p < 0.05.

As to texture features, BPL increased HGZE by 13.9% and decreased LGZE 13.3% compared to OSEM on average with statistical significance (p<0.05). Other texture features did not represent statistical differences between OSEM and BPL images (**Table 2**). Changes of HGZE or LGZE with BPL did not correlate with SUVmax, SUVpeak, or MTV in OSEM. They did not correlate with changes of SUVmax, SUVpeak, or MTV with BPL, either.

**Figure 2:**
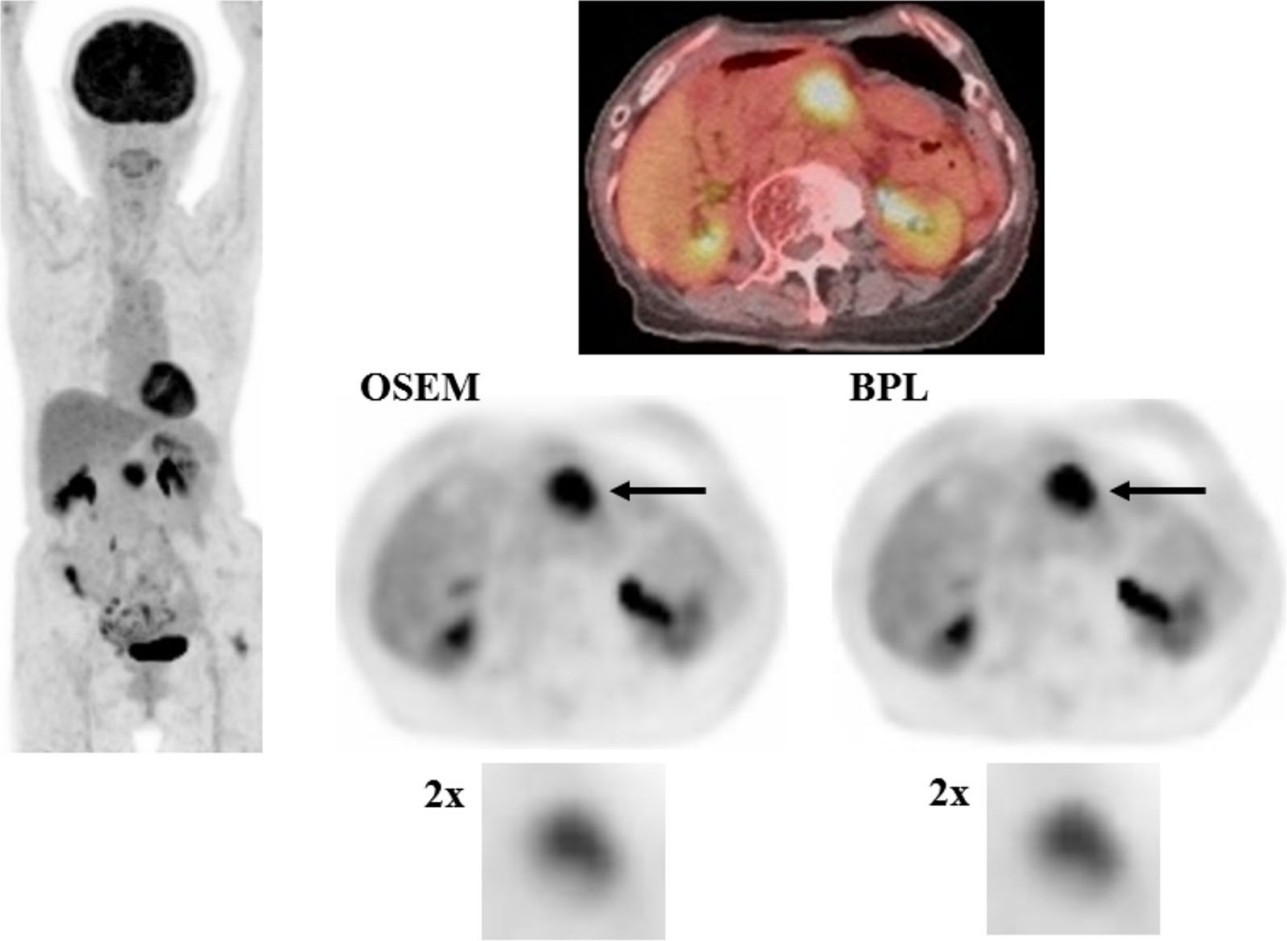
A case with pancreatic cancer (larger lesion). FDG uptake corresponding to the pancreatic cancer lesion (arrow) was visualized almost equally in OSEM and BPL images (right, middle and lower). SUVmax in OSEM (9.3) was similar to that in BPL image (9.2), and MTV was smaller in OSEM (10.3 ml) than in BPL image (10.9 ml). BPL increased high gray-level zone emphasis by 7.6% and decreased low gray-level zone emphasis by 33.0% compared to OSEM. (Left: maximum intensity projection image of FDG PET; right upper: fused FDG PET/CT image; right middle: FDG PET images reconstructed with OSEM and BPL algorithms; right lower: images focusing on tumor FDG uptake magnified by a factor of 2).

A case with pancreatic cancer is presented in **Figure 2**, in which no significant differences were observed between OSEM and BPL images.

### The Effect of BPL According to SUVmax in OSEM

There were 47, 64, and 42 lesions with low, intermediate, and high SUVmax in OSEM, respectively. The increase of SUVmax with BPL was observed in 34 (72%), 27 (42%), and 9 (21%) lesions with low, intermediate, and high SUVmax in OSEM, respectively.

**Figure 3:**
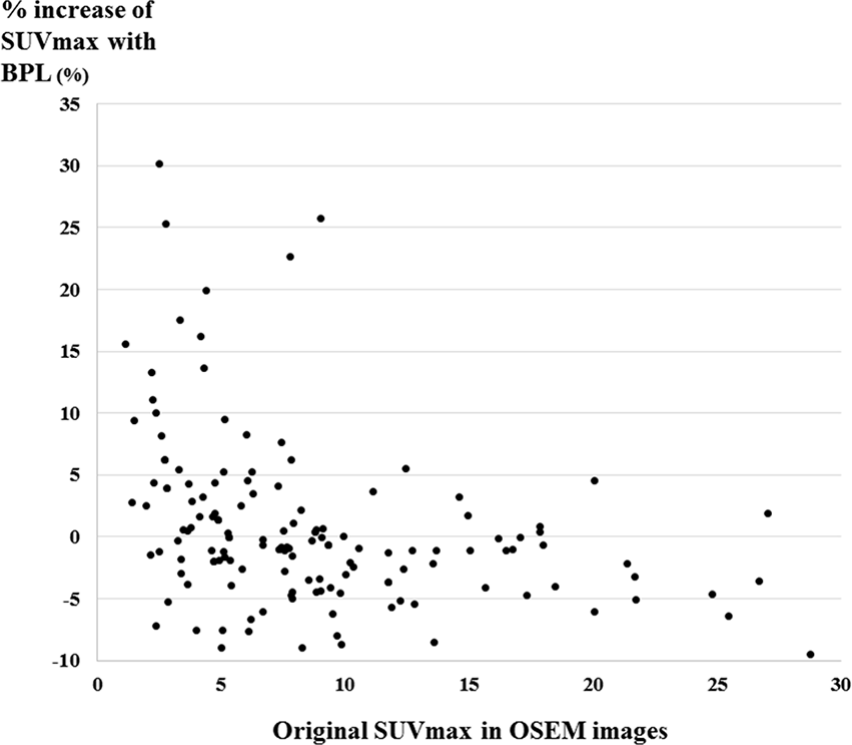
The distribution of %increase of SUVmax with BPL *versus* original SUVmax in OSEM images shown as a scatter plot. The increase of SUVmax with BPL was observed in 72% (34/47), 42% (27/64), and 21% (9/42) of low, intermediate, and high SUVmax in OSEM images, respectively. (Low, intermediate, and high SUVmax were defined as SUVmax less than 5, 5 to 10, and more than 10 in OSEM images, respectively).

The distribution of %increase of SUVmax with BPL *versus* original SUVmax in OSEM is shown as a scatter plot in **Figure 3**. BPL increased and decreased SUVmax significantly in lesions with low and high SUVmax in OSEM images, respectively (p < 0.01 and p<0.0001). No significant increase of SUVmax with BPL was observed in lesions with intermediate SUVmax in OSEM images.

## Discussion

In this study we evaluated the effects of BPL on lesions in the upper abdominal region, namely, metastatic liver cancer and pancreatic cancer. Only limited data have been available so far with regard to the BPL effects focusing on abdominal lesions. As far as we acknowledge, there has been only one published paper of FDG PET imaging dealing with this topic in metastatic liver cancer (7).

The effect of BPL was prominent in small lesions in this study. BPL improved visual scores and increased SUVmax compared to those in OSEM images. These results agreed with previous reports on small pulmonary nodules (2, 4, 5). Small liver or pancreatic lesions are often not visualized clearly in FDG PET examinations, while BPL improved visual conspicuity in one-third of such small lesions in this study. The results obtained in this study warrant further studies to determine whether FDG PET images with BPL offer better diagnostic utility than those with OSEM in clinical practice.

Small lesions tend to represent relatively low FDG uptake compared with large lesions generally. In this study, the mean SUVmax with OSEM was 4.5 in small lesions and 10.1 in larger (greater than 2 cm) lesions. The increase in visual score with BPL showed statistically negative correlation with SUVmax in OSEM images in small lesions. SUVmax-based analysis also revealed that %increase of SUVmax with BPL showed statistically negative correlation with SUVmax in OSEM images. These results indicated that the effect of BPL was observed greatly in lesions with low SUVmax. The BPL effects on small lesions observed in this study were considered to be closely related to the relatively low FDG uptake in small lesions. These observations were supported partly by the evidence that BPL is effective in low-count clinical PET images (13, 14). The point spread function was used in both BPL and OSEM reconstruction and hence would not have been an explanation for the increased SUVmax and conspicuity of small lesions in this study.

In lesions more than 2 cm in greatest diameter, BPL decreased SUVmax and increased SUVpeak and MTV. However, none of them showed statistical differences. BPL was demonstrated to affect texture features as well. Notably, BPL increased HGZE and decreased LGZE with statistical significances. HGZE was reported to correlate negatively with LGZE (12) and to be higher when a tumor has multiple scattered foci of intense FDG uptake (15). HGZE was also reported to increase when the blurring effect of respiratory motion decreases in a tumor (16).

The beta value 700 was determined according to our previous study of PET image quality with BPL reconstruction algorithm using several beta values (10). This value is higher than that reported in earlier studies (1), but is similar to that used in recent studies (5, 13). Although Te Riet J et al. reported that PET images with beta value 700 slightly compromised lesion detectability and SUV recovery, this study demonstrated that improvement of visual conspicuity and increase of SUVmax with BPL were observed in small upper abdominal lesions. Similar values of quantitative metrics, except for a few texture features, were observed between OSEM and BPL images in larger lesions. The beta value 700 may not be ideal in some instances but is considered acceptable in clinical situations and thus used in our hospital.

This study had some limitations. First, upper abdominal malignant lesions were limited to metastatic liver cancer and pancreatic cancer. We selected these cancers as they were commonly examined with FDG PET/CT, and clear visualization of them was often difficult due to respiratory motion. Other upper abdominal malignant lesions, such as hepatocellular carcinoma, bile duct cancer, or intraductal papillary mucinous carcinoma of the pancreas, may be interesting as next research targets to clarify the BPL effects. Second, this study focused only on comparison of visual and quantitative results in upper abdominal lesions between OSEM and BPL images. The effect of BPL on lesion detection was not evaluated. Further studies are desirable to clarify the advantages of FDG PET images with BPL, especially in small upper abdominal malignant lesions, in clinical practice. This study was conducted in a retrospective manner. This may also be included as a limitation.

## Conclusion

This study demonstrated that BPL improved conspicuity of small or low-count upper abdominal malignant lesions in clinical FDG PET/CT examinations. Only two TFs represented significant differences between OSEM and BPL images of all quantitative metrics in larger lesions. Larger studies are warranted to validate these findings in clinical situations.

## Data Availability Statement

The raw data supporting the conclusions of this article will be made available by the authors, without undue reservation.

## Ethics Statement

The studies involving human participants were reviewed and approved by Osaka University Hospital. Written informed consent for participation was not required for this study in accordance with the national legislation and the institutional requirements.

## Author Contributions

The study was designed by MT, TK, and JU. All authors participated in collecting data. MT and FS prepared the manuscript and contributed to data analysis and interpretation. All authors contributed to the article and approved the submitted version.

## Conflict of Interest

The authors declare that the research was conducted in the absence of any commercial or financial relationships that could be construed as a potential conflict of interest.

## Publisher’s Note

All claims expressed in this article are solely those of the authors and do not necessarily represent those of their affiliated organizations, or those of the publisher, the editors and the reviewers. Any product that may be evaluated in this article, or claim that may be made by its manufacturer, is not guaranteed or endorsed by the publisher.
